# Genomic Context and Insert Orientation in the Regulation of Transgene Expression in Adenoviral Vectors

**DOI:** 10.3390/ijms27062542

**Published:** 2026-03-10

**Authors:** Anna Muravyeva, Svetlana Smirnikhina

**Affiliations:** Laboratory of Genome Editing, Research Centre for Medical Genetics, Moskvorechye, 1, 115522 Moscow, Russia

**Keywords:** adenoviral vectors, transgene expression, genomic context, insertion orientation, endogenous adenoviral promoters, major late promoter (MLP), E3 promoter, E1A enhancer, gene therapy

## Abstract

Adenoviral vectors are among the most efficient platforms for gene delivery; however, the level and pattern of transgene expression in these vectors are largely shaped by the viral genomic context. This review discusses the mechanisms of adenoviral transcription and alternative splicing and how they influence the expression of inserted expression cassettes. Particular attention is given to the role of insertion orientation and transgene placement within the E1 and E3 regions, as well as to the effects of viral regulatory elements, including the E1A enhancer. We analyze evidence on the use of insulating sequences to reduce nonspecific activation and improve the controllability of transgene expression. We also consider the use of endogenous adenoviral promoters—the major late promoter (MLP) and the E3 region promoter—and their contribution to enhanced transgene expression through late viral transcription. Overall, these findings support principles for the rational design of adenoviral vectors, both for high-level protein production and for building systems with regulated or tissue-specific expression.

## 1. Introduction

Adenoviruses are non-enveloped DNA viruses of the family *Adenoviridae* (genus *Mastadenovirus*) characterized by an icosahedral capsid ~70–100 nm in diameter and a linear double-stranded DNA genome of ~36 kb [1,2,3]. A key feature of adenoviruses is their ability to remain largely episomal rather than integrating into the host-cell genome, which makes them particularly attractive for the development of vector systems for gene delivery [4,5]. Accordingly, adenoviral vectors have been engineered, most commonly based on human adenovirus type 5, as the best-studied serotype and thus the most widely used platform [2,6,7].

Adenoviral vectors offer several important advantages. First, they can transduce both dividing and non-dividing cells, enabling their application across a broad range of diseases. Second, owing to their broad tropism, these vectors often achieve high delivery efficiency [8,9,10]. Viral entry is primarily mediated by the coxsackie and adenovirus receptor (CAR), which is expressed in many tissues, further expanding the potential scope of use [11,12,13,14,15]. Another major advantage is their high packaging capacity: in first-generation vectors, it is ~7.5 kb, whereas in helper-dependent (gutless) systems, it can approach 36 kb—close to the full length of the wild-type adenoviral genome [16,17]. This makes adenoviral vectors preferable to, for example, adeno-associated viruses (AAVs), whose ~4.7 kb genome often limits delivery of multi-component constructs required for modern gene therapy [18,19,20]. In practice, adenoviral vectors are widely used in oncolytic therapy and vaccine development [21]. Approved adenovirus-based therapeutics include Gendicine [22] and Oncorine [23], authorized in 2003 and 2006, respectively, for the treatment of head and neck cancers, leveraging immune activation mechanisms. Examples of adenoviral vaccines include Sputnik V [24], Vaxzevria (AstraZeneca) [25] and Convidecia (CanSino Biologics), which are based on human or animal adenoviruses [26,27].

Most contemporary adenoviral vectors belong to the first generation and carry deletions in the E1 and E3 regions, which are expressed early in the viral life cycle. Deletion of E1, which is required for viral DNA replication, renders the vector replication-defective [28,29,30]. Deletion of E3, which encodes proteins that inhibit host immune responses, reduces viral immunogenicity and provides additional space for transgene cloning [31,32,33,34]. These vectors are propagated in E1-complementing cell lines such as HEK293 [6]. In addition to first-generation vectors, second-generation adenoviral vectors (with additional deletions in E2 and/or E4 [35,36]) and third-generation helper-dependent vectors, which are largely devoid of viral coding sequences [37,38,39], have also been developed; however, in practice, they are used far less frequently due to the complexity of production and scale-up. By contrast, numerous commercial systems and ready-to-use platforms are available for first-generation vectors, substantially simplifying their application in both research and translational settings [40,41,42]. Accordingly, first-generation adenoviral vectors remain the mainstay for both basic studies and the development of commercial products in gene therapy and vaccinology. 

**Figure 1:**
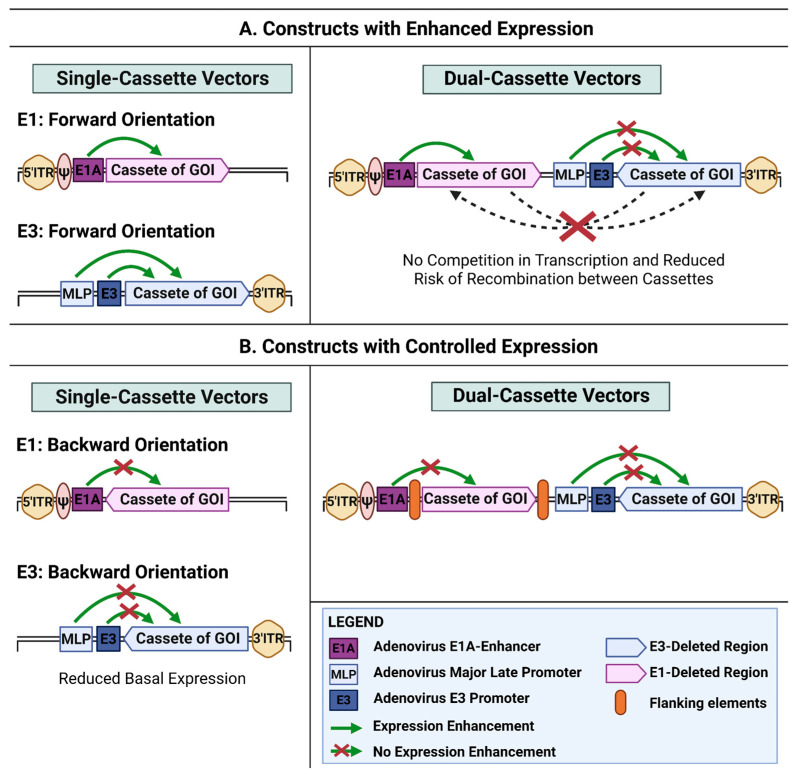
Insertion orientation and genomic context as regulators of transgene expression in adenoviral systems. The GOI cassette contains its own heterologous promoter and a poly(A) signal. This figure was created by using https://www.biorender.com (accessed on 15 February 2026).

A key design decision for such vectors is the choice of genomic locus and the orientation of the expression cassette. In most cases, transgenes are cloned into the deleted E1 and/or E3 regions, and the cassette can be inserted in either the forward or reverse direction relative to viral transcription. This may affect transgene expression levels, packaging efficiency, viral titer, and genetic stability [43]. The orientation of multiple transgenes in bicistronic or dual-cassette vectors is likewise important, particularly with respect to their interactions with endogenous viral promoters (e.g., the major late promoter, MLP, and the E3 promoter). This review summarizes key approaches to the design of adenoviral vectors carrying one or two expression cassettes, with a focus on how transgene orientation and interactions with viral regulatory elements influence vector assembly efficiency, viral titer, and the level and stability of transgene expression. A summary of this article is shown in Figure 1.

## 2. Transcriptional Organization of the Adenoviral Genome

### 2.1. Adenoviral Transcription Units

Understanding the transcriptional architecture of the adenoviral genome and the regulation of viral gene expression is essential for the development of adenoviral vectors. Owing to the well-characterized structure of adenoviral transcription units and the extensive use of alternative splicing, adenoviruses have become one of the most widely used platforms for genetic cargo delivery. Genome engineering through targeted deletions increases the packaging capacity available for transgenes, enabling the use of these vectors in diverse gene therapy applications, including gene editing and gene replacement therapies [44,45].

The adenoviral infectious cycle is tightly orchestrated and comprises early and late phases [46,47]. This division reflects both the temporal sequence of events during infection and the stepwise activation of specific transcription units, each encoding proteins required at defined stages of viral replication. During the early phase, the early genes E1A, E1B, E2 (E2A and E2B), E3, and E4 are expressed to prime the host cell for viral replication [48]. These gene products activate or modulate cellular transcription factors, suppress apoptosis, and ensure efficient replication of viral DNA (Table 1).

**Figure 2:**
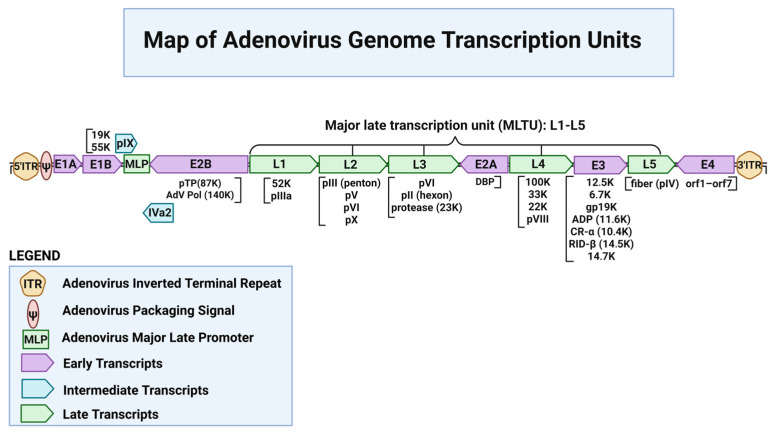
Map of adenovirus genome transcription units. A more detailed description of the transcription units of the adenoviral genome is provided in the text (Table 2). This figure was created by using https://www.biorender.com (accessed on 13 February 2026).

After completion of viral DNA replication, the major late promoter (MLP) is activated, initiating the late phase of infection, during which the L1–L5 genes encoding viral structural proteins are expressed [76]. Although all late mRNAs are transcribed from a single promoter, transcript diversity is generated through cascade alternative splicing and the use of multiple polyadenylation sites [77]. This results in the production of numerous mature mRNAs, each containing a common 5′ leader sequence (the tripartite leader, TPL) that enhances translation [78,79]. The L1–L5 transcription units include the hexon (L3) gene encoding the major capsid protein, penton base and fiber (L2 and L5) involved in viral attachment and entry, packaging proteins, and late regulatory factors such as L4-22K and L4-33K, which contribute to splicing regulation and virion assembly [80,81,82,83]. Each transcription unit produces multiple mRNA species via alternative splicing from a single pre-mRNA, as illustrated in Figure 2. In this review, terms such as forward/reverse or parallel/antiparallel refer to the orientation of the expression cassette relative to the direction of viral transcription along the Ad5 genome (Figure 2). In the E1 locus, these configurations are often denoted as E1L and E1R, indicating cassette orientation toward the left or right genomic direction, respectively. Because transcripts produced from the major late transcription unit can extend across large portions of the viral genome, insertions in the E3 locus may be exposed to read-through transcription and alternative splicing, which in some cases can influence transgene expression or contribute to cassette instability.

### 2.2. Alternative Splicing of Adenoviral Transcripts

Adenoviruses employ a highly developed alternative splicing program that enables a broad spectrum of mRNA species to be generated from a limited number of transcription units. This is achieved through tightly regulated transcription initiation, the presence of multiple donor and acceptor splice sites, and the coordinated activity of viral and host factors. Such transcript diversity allows the virus to precisely coordinate protein expression at different stages of replication and to adapt to conditions within the host cell (Table 2).

**Table 2:** Key Features of Alternative Splicing in Adenoviruses.

Region	Transcription Unit	Promoter	Expression Timing	Key mRNAs	Splicing Features	Reference
E1	E1A	P_E1A	Earliest	13S, 12S, 9S (major); 10S, 11S (minor)	These mRNAs share common 5′ and 3′ ends but differ in intron length. Early in infection, 13S and 12S predominate, whereas 9S accounts for <5% of total E1A mRNA; at late times, 9S becomes dominant. Initiate infection.	[84,85,86]
	E1B	P_E1B	Early	22S, 13S (major); 14.5S, 14S (minor)	Splicing of one or two introns from a common precursor. The ratio of major mRNAs changes over the course of infection; at late times, 13S accumulates ~20-fold more than 22S. Produce mRNAs for the 19K and 55K genes.	[84,87]
Intermediate region (between E1B and MLP)	IX	P_IX	Intermediate	IX	No splicing; encoded by a separate single-exon transcript, not spliced to the tripartite leader; the mRNA encodes one protein. Structural capsid component.	[88]
Intermediate region (between E2 and MLP)	IVa2	P_IVa2	Intermediate	IVa2	Spliced mRNA derived from multiple transcripts. IVa2 acts as a transcriptional activator that enhances MLP activity and participates in DNA packaging into the capsid (binds the packaging signal ψ and is required for completion of virion assembly).	[89,90]
E2	E2A	P_E2A (l-strand)	Early	DBP	Among multiple E2A mRNAs, one dominant species encodes the DNA-binding protein (DBP) and is generated after removal of two introns.	[91,92]
	E2B	P_E2B (l-strand)	Early	pTP, DNA polymerase	E2B transcripts bypass the E2A mRNA polyadenylation signal and extend to a downstream poly(A) site. They encode the preterminal protein pTP (87K) and adenoviral DNA polymerase (Adv-Pol, 140K), required for viral DNA replication.	
E3	E3	P_E3 + MLP	Early	~9 mRNAs	Multiple mature mRNA isoforms generated via alternative splicing. At least 9 E3 mRNAs arise from two precursors (E3A and E3B) with a shared cap but different 3′ ends and polyadenylation sites. E3B has one poly(A) site, whereas E3A has four. E3 proteins (12.5K, 6.7K, gp19K, 11.6K (ADP, adenovirus death protein), 10.4K (CR-α, cytokine receptor), 14.5K (RID-β, receptor internalization and degradation complex), and 14.7K) mediate host immune evasion.	[93,94]
E4	E4	P_E4 (l-strand)	Early	Up to 24 mRNAs with identical 5′ and 3′ ends; orf1–orf7	A single primary transcript is spliced into up to 24 distinct mRNAs with identical 5′ and 3′ ends. The region contains 7 ORFs; proteins from most ORFs are detected in infected cells except ORF3/4. Involved in regulation of transcription and replication.	[95,96]
UXP	UXP	P_UXP (l-strand)	Late	UXP	No splicing; the mRNA encodes a single protein. Putatively involved in DNA replication.	[97,98]
MLTU (L1–L5)	MLTU	MLP	Late	>20 mRNAs from one promoter, polyadenylated at five sites to form L1–L5 families	All contain the common 5′ tripartite leader (TPL), 201 nt, consisting of three leader exons (LI, LII, LIII). Some also include an additional i-leader (440 nt). Complex alternative splicing: TPL is joined to different combinations of L1–L5 exons. Encodes structural components and splicing regulators.	[76,99]
	L1	MLP	Late	2 mRNAs (pIIIa, 52K)	The 52K-encoding mRNA is produced in both phases, whereas pIIIa mRNA appears only late. Two poly(A) sites. Contains alternative exons for 52K and pIIIa (different acceptors).	[100,101]
	L2	MLP	Late	4 mRNAs (pIII, pV, pVII, pX)	A shared polyadenylation site for four mRNAs encoding pIII (penton base), pV (major core protein), pVII (core protein), and pX.	[99,102,103]
	L3	MLP	Late	3 mRNAs (pVI, pII, 23K)	L3 pre-mRNA is spliced into three major mRNAs with a common poly(A) site encoding pVI (hexon-associated protein), pII (hexon), and the 23K viral protease.	[101,104]
	L4	MLP	Late	4 mRNAs (L4-22K, L4-33K, L4-100K, pVIII)	L4-100K and L4-22K are transcribed at the beginning of the late phase; L4-22K is driven by an internal promoter within the L4-100K ORF. L4-22K represses early gene expression and activates L1–L5 transcription. L4-33K is a viral alternative splicing factor that promotes splicing of late transcripts with weak 3′ sites and regulates their accumulation. pVIII is a minor capsid protein that stabilizes the virion and contributes to packaging by mediating interactions between viral DNA and the capsid shell.	[81,105,106]
	L5	MLP	Late	1 mRNA (pIV)	Encodes only pIV (fiber). These transcripts exhibit unique combinations of 5′ leader sequences.	[103,107]

Thus, understanding the organization of adenoviral transcription units, their splicing patterns, and the distribution of regulatory elements is essential for rational adenoviral vector design. This knowledge enables more informed selection of the insertion site and orientation of expression cassettes, helps anticipate interactions with viral regulatory sequences, and supports further genome engineering (e.g., additional deletions) to increase packaging capacity when large transgenes need to be accommodated.

## 3. Effects of Insertion Orientation and Viral Regulatory Elements on Transgene Expression

### 3.1. Single-Cassette Adenoviral Vectors

Single-cassette adenoviral vectors are constructs in which a single transgene expression cassette is inserted into the viral genome, most commonly into the E1 region (and less frequently into E3), which is deleted in first-generation vectors. These constructs are widely used in research because they provide the simplest route for cloning transgenes into an adenoviral vector. An analysis of the relevant literature indicates that even when an identical expression cassette is used, the orientation of insertion within the viral genome can substantially affect transgene expression levels, viral titers, and packaging efficiency, making these parameters an important consideration for optimizing adenoviral vector design.

For the E1 region, orientation effects appear to be less uniform and are largely determined by the specific transgene and promoter. In E1, differences between the E1L and E1R orientations in terms of viral titer, expression output, and mRNA and protein levels were inconsistent and depended on the particular transgene and promoter used [108]. Overall, vectors with insertions in E1L/R (as compared with E3 and E4) tended to yield higher titers and expression levels and remain the most common due to the practical convenience of vector construction. In a study aimed at optimizing first-generation vectors for gene therapy and vaccine development, transgene orientation in E1 was also shown to significantly affect viral growth characteristics [109]. In particular, vectors with the cassette inserted in the forward orientation in E1 displayed advantages in titer and genetic stability during extended passaging (up to 21 passages). Similarly, another study reported that a transgene cloned in the forward direction into the E1 locus produced higher and earlier expression across multiple cell lines, with only minor differences in the onset of expression [110]. The orientation of the expression cassette replacing E1 in the vector backbone had a significant effect on the level of transgene expression, with vectors containing expression cassettes directed towards the right end of the Ad genome exhibiting up to 7-fold higher β-galactosidase activity in infected cells than those with inserts in the opposite orientation [111]. It has been suggested that transcription factors binding the E1A enhancer may boost the activity of a heterologous promoter when the insert is in the forward orientation. Thus, even after deletion of the E1 coding sequences, regulatory elements in this region can continue to influence transgene expression.

A key explanation for orientation-dependent effects in E1 is the residual activity of the E1A enhancer retained in the vector after E1 deletion, which may enhance transcription of nearby cassettes when their orientation is aligned [29,30]. This element is part of the left early transcriptional regulatory region and contributes to the initiation of E1A transcription soon after viral DNA enters the cell nucleus [112,113]. It contains binding sites for cellular transcription factors and can therefore enhance not only the native E1A promoter but also heterologous promoters located nearby within recombinant vectors [114,115]. Mechanistically, these effects may arise through several distinct processes, including enhancer-mediated transcriptional activation by regulatory elements within the E1A region, transcriptional read-through from adjacent viral transcription units into the transgene cassette, or incorporation of the transgene transcript into adenoviral splicing pathways.

In the E3 region, by contrast, orientation effects are considerably more pronounced. Several independent studies have shown that cassettes oriented in the direction of E3 transcription and late MLTU transcripts more often support earlier and/or higher expression. In models expressing the vesicular stomatitis virus glycoprotein gene (*VSV-G*) [116] and the hepatitis B virus surface antigen gene (*HBsAg*) [117], expression in the forward orientation began at early stages of infection (concurrent with the E2 72K protein) and did not always require viral DNA replication. This, in turn, points to a major contribution of the endogenous early E3 promoter, which is active early in infection, and the MLP, which drives transcription through this region after viral DNA replication [99,118]. Deletion of a splice site within the E3 region was also reported to increase *HBsAg* expression during the late phase [117]. Thus, insertion orientation and interactions with endogenous promoters influence transcriptional output, and reverse insertion may be useful when expression needs to be constrained. Overall, these observations suggest that orientation relative to the direction of viral transcription influences the timing of transgene expression in the E3 locus. Inserts aligned with the direction of viral transcription may be expressed early through read-through transcription from upstream units and later through major late promoter activity following viral DNA replication, whereas inserts in the opposite orientation tend to show weaker or delayed expression.

Conversely, in an antiparallel orientation, expression was weaker and more often depended on the late phase and activation of the MLP. In a study using the HSV-1 *gB* gene, a cassette inserted in the reverse orientation in E3 provided little to no early expression in several cell lines, whereas the vector with a forward-oriented insert replicated in all tested lines and produced high-level *gB* expression [119]. Moreover, the heterologous SV40 promoter was shown to function not as an autonomous promoter but rather as a cryptic splice acceptor for viral transcripts initiated in upstream 5′ regions of the genome [120,121].

Taken together, these findings support several general conclusions for the design of single-cassette adenoviral vectors. First, E1 remains the most suitable locus for single-cassette constructs. This is because insertion into E1 more often yields high titers, passaging stability, and robust expression levels, while orientation plays a modifying rather than a determining role [108,111]. Second, the E3 region can also be used for transgene insertion, but—as multiple studies indicate—requires more careful selection of cassette orientation. A forward-oriented transgene aligned with viral transcription can “hitchhike” on endogenous promoter activity and the MLP to achieve high expression, whereas an antiparallel insertion can serve as a tool to modestly limit expression or shift it toward the late phase of infection [116,117,119].

### 3.2. Dual-Cassette Adenoviral Vectors

Unlike single-cassette constructs, dual-cassette adenoviral vectors create a markedly more complex transcriptional environment because two independent expression units—often driven by strong heterologous promoters—coexist within the same viral genome. Under these conditions, cassette orientation, their relative arrangement, and their placement in distinct genomic regions (E1, E3, E4) become key determinants of vector performance rather than secondary design choices. Available evidence indicates that dual-cassette designs are more prone to transcriptional interference, promoter competition, and modulation by adenoviral regulatory elements—effects that are typically much less pronounced in single-cassette vectors.

Several studies have highlighted that the E3 region is particularly sensitive to the orientation of a second expression cassette. When a cassette under strong promoters was inserted into E3 in the forward orientation (i.e., aligned with viral transcription), viable recombinant clones were often difficult or impossible to recover, likely reflecting reduced efficiency of viral particle assembly and production of infectious virions. In contrast, inserting the same cassette into E3 in the reverse orientation, or placing it in E1 in the forward orientation, allowed the generation of infectious viruses. In practice, genetically stable vectors with robust expression of both transgenes were obtained with a configuration combining a forward insertion in E1 and a reverse insertion in E3 [122]. It has also been reported that sharing identical regulatory elements between the two cassettes (e.g., introns or enhancers) can measurably alter transcript abundance and protein output. These observations are commonly attributed to transcriptional interference between heterologous promoters and endogenous regulatory elements within E3 and the major late transcription unit (MLTU), as well as to potential perturbation of normal splicing patterns of late viral transcripts [123,124]. 

With identical cassette designs, no major differences in in vivo expression levels of secreted reporter proteins were observed across dual-cassette adenoviral vectors constructed using different locus combinations (E1/E3, E1/E4) [125]. Thus, the locus itself may not be the limiting factor when promoters and insertion orientations are the same. However, stability profiling revealed a consistent vulnerability of E3-based inserts: during serial passaging, cassettes placed in E3 were more likely to undergo deletions, whereas inserts in other regions remained stable even at later passages [125]. This supports the view that E3—being involved in late transcriptional programs and alternative splicing—provides a less stable platform for the long-term expression of large inserts.

The most informative contrasts in both stability and expression were obtained when comparing (i) strategies placing two expression cassettes within the same E1 locus and (ii) spatially separating the cassettes between E1 and E3 (typically forward in E1 and reverse in E3) [126]. Comparative analyses of co-expression formats showed that vectors expressing two genes from a single cassette via a 2A peptide, as well as bidirectional expression designs within E1, could be efficiently rescued and amplified and remained genetically stable. However, 2A-based constructs tended to yield lower transgene output than matched controls with separate expression, whereas bidirectional E1 designs achieved comparable levels. The highest overall stability combined with strong expression was reported for vectors carrying two independent tandem cassettes within E1; notably, expression of the cassette positioned closer to the left ITR exceeded that of the downstream cassette [126]. This asymmetry may reflect local enhancer effects of the left ITR and the residual E1A enhancer region on nearby promoters [127,128]. Promoter interference is another plausible contributor, as two strong promoters placed in close proximity can impair each other’s activity [129,130]. Thus, even within a single E1 locus, cassette order and orientation can shape the expression balance between the two genes. Although the difference in expression was modest, inclusion of short spacer sequences between adjacent cassettes has been proposed to reduce promoter cross-talk and equalize expression levels [131,132].

#### Homologous Recombination Between Cassettes and Genetic Instability of Adenoviral Vectors

Discussion of cassette orientation and genomic placement naturally leads to another key design parameter of dual-cassette adenoviral vectors: structural homology between the expression cassettes. While this issue is most evident in dual-cassette vectors, it may also be relevant for single-cassette designs that contain repeated regulatory elements. Multiple studies have shown that incorporating identical or highly homologous promoters, enhancers, and poly(A) signals within the same vector genome markedly increases the risk of homologous recombination and, consequently, cassette deletions.

In studies assessing multi-transgene expression in an rAd35-based vector, the endogenous E3-region promoter was found to support transgene expression levels comparable to those driven by the heterologous CMV promoter, but only in E1-complementing cells; in a non-complementing cell line, E3 promoter activity was reduced by approximately 10–100-fold relative to CMV [133]. Use of two identical CMV–MCS–SV40pA cassettes inserted into the E1 and E3 loci resulted in genome instability during serial passaging due to homologous recombination, predominantly affecting the E3 insertion. Replacing one poly(A) signal eliminated this instability while preserving high expression, underscoring the importance of minimizing homology between cassettes for stable co-expression. Similar observations were reported for Ad5 vectors developed for clinical testing in ovarian cancer gene therapy [134]. Two identical expression units driven by CMV promoters and terminated by SV40 poly(A), inserted in E1 in the forward orientation, promoted homologous recombination and deletion of one of the expression units. Substituting regulatory elements with non-homologous counterparts restored independent expression of both transgenes and improved genetic stability, indicating that the underlying problem is structural—driven by cassette identity rather than insertion site.

The literature suggests that, in dual-cassette adenoviral vectors, orientation becomes a system-level factor that affects not only expression output but also the ability to recover viable viruses. The E1 locus is generally considered a more stable platform, where forward-oriented inserts often yield higher expression and improved stability, potentially due to residual effects of the E1A enhancer. In contrast, the E3 region is sensitive to transcriptional direction: forward orientation may enhance expression but increases the likelihood of transcriptional interference and genetic instability, whereas reverse orientation can mitigate these risks at the cost of reduced expression. Importantly, placing two identical cassettes into different regions does not prevent deletions, because homologous recombination likely occurs not within a single viral genome, but between distinct copies of viral DNA during replication in an infected cell. As adenoviral DNA accumulates to high copy numbers in the nucleus, conditions favor intergenomic crossover between homologous segments, resulting in shorter, selectively advantageous genomes that have deleted one of the repeated cassettes [135,136,137,138,139]. Therefore, a central strategy to ensure the stability of dual-cassette adenoviral vectors is to minimize homology between regulatory elements, alongside the rational choice of insertion locus and cassette orientation. Various regulatory elements have been explored to moderate enhancer interference in adenoviral vectors, including transcriptional terminators and chromatin insulating elements such as ubiquitous chromatin opening elements [140,141].

Overall, several practical strategies can be used to mitigate these issues during vector design:

- using non-homologous poly(A) signals to reduce recombination between transcription units;
- employing different promoters for multiple expression cassettes;
- introducing spacer or insulator sequences between transcription units;
- minimizing repeated regulatory elements such as identical introns or enhancers;
- sequence diversification of regulatory regions to reduce homologous recombination.

### 3.3. Effects of Insertion Orientation on Regulated Transgene Expression

Regulated expression of therapeutic genes is a central goal of modern gene therapy. The ability to control the level, timing, and tissue specificity of expression can substantially improve the safety of vector systems, particularly when the transgene product is cytotoxic, highly bioactive, or immunogenic. Such applications include adenoviral vectors carrying “suicide” genes for cancer therapy [142], locally produced cytokines and growth factors [143,144], as well as targeted correction of genetic defects in specific cell types. However, even when tissue-specific or inducible promoters are used, transgene expression profiles in adenoviral vectors may deviate markedly from expectations. This is largely due to the influence of endogenous regulatory elements within the adenoviral genome, making cassette locus and orientation key determinants of both the level and pattern of expression.

Several studies have shown that cassette orientation in adenoviral vectors primarily affects basal (uninduced) expression, which is critical when working with toxic or immunologically active genes. In dual-cassette Tet-ON/Tet-OFF vectors, changing the orientation of the transcriptional activator cassette within E3 had little effect on doxycycline-induced expression, but substantially reduced basal activity when the cassette was inserted in the reverse orientation, as measured via reporter gene expression in the absence of doxycycline, likely by attenuating the impact of adjacent viral regulatory elements [145]. In more complex designs combining two independent inducible systems (the streptogramin-responsive PIP system and the tetracycline-responsive TET system), orientation effects in the E1 locus were even more pronounced: reverse orientation of the activator cassette led to loss of control in the TET system and substantial leakiness in the PIP system, likely driven by excessive expression of the PIP activator as the first cistron of the cassette. In contrast, the forward orientation supported balanced and tightly regulated performance of both systems [146]. Thus, cassette orientation influences not only interactions with viral regulatory elements but also intravector transcriptional cross-talk between neighboring promoters and polycistronic designs.

Orientation becomes particularly important in the context of interactions between inserted promoters and adenoviral enhancers. When tissue-specific or inducible promoters are cloned into the E1 locus, their activity can be substantially distorted by the E1A enhancer: forward orientation is often associated with enhanced expression accompanied by loss of tissue specificity and increased basal activity, whereas reverse orientation can partially mitigate these effects. Moreover, positioning cassettes near the right ITR in either orientation has been reported to provide more stable selectivity and lower nonspecific expression than cloning into E1 [147]. A similar impact of the E1A enhancer in an adenoviral vector altered both tissue specificity and activity of a transcription-gene promoter [148]. Other studies examining tissue-specific promoters in adenoviral vectors have suggested that the effect of the E1A enhancer may depend on the promoter type and cellular context. For example, in an adenoviral vector system lacking the E1A enhancer, the albumin promoter supported tissue-specific expression in hepatoma cells but drove relatively low transcriptional activity. In the presence of the E1A enhancer, however, transcription from the albumin promoter increased approximately 5-fold [149]. By contrast, no comparable stimulatory effect of the E1A enhancer on an immunoglobulin promoter was detected in myeloma cells. Together, these findings indicate that the regulatory impact of the E1A enhancer is both tissue- and promoter-dependent, likely reflecting cell-type-specific repertoires of transcription factors.

To address loss of selectivity caused by enhancer effects or cryptic transcription initiation sites within the adenoviral genome, insulating elements flanking the expression cassette and containing transcription termination signals have been proposed. Such insulator sequences effectively blocked undesired transcriptional activation and preserved high selectivity of *HSVtk* expression from a forward-oriented cassette in the E1 locus, restricting expression to target cells carrying the *ERBB2* oncogene [150]. This is particularly relevant for “suicide gene” approaches in cancer gene therapy. Shielding a transgene in the E1 locus by flanking the cassette on both sides markedly reduced basal expression and increased inducibility (by ~40-fold in vitro and ~230-fold in vivo based on measured hAAT expression levels). Vectors lacking insulator sequences showed substantially weaker induction, with induction ratios approximately 40-fold lower in vitro and ~15-fold lower in vivo. The strongest effect was observed when the transgene was inserted in the reverse orientation in E1, whereas forward orientation resulted in high non-inducible activity [127]. These results are consistent with the proposed influence of the viral E1A enhancer on nearby cassettes and are supported by other studies. For instance, in adenoviral vectors carrying the radiation-inducible EGR-1 promoter in the E1 locus in the forward orientation, substantial nonspecific expression was observed in the absence of irradiation. Incorporation of a bGH poly(A) signal as an insulating element—placed upstream of the cassette or on both sides—reduced nonspecific cytotoxicity by 1.6–2.3-fold with minimal impact on induced expression [151].

In summary, cassette orientation and genomic placement are key determinants of transgene expression profiles in adenoviral vectors. In the vicinity of the packaging signal, forward-oriented cassettes can exhibit a pronounced enhancer effect associated with E1A elements, resulting in higher expression relative to placement near the right ITR; this effect appears replication-independent and is consistent with promoter interference. Forward orientation is therefore well suited for achieving high expression when strict selectivity is not required. In contrast, for inducible or tissue-specific expression, alternative insertion sites (E3/E4) and reverse orientation are often preferable, as they reduce basal activity. Additional stabilization can be achieved by flanking the cassette with insulating elements containing poly(A) signals, which limit the influence of viral enhancers and cryptic transcription initiation sites.

## 4. Enhancement of Transgene Expression by Endogenous Adenoviral Regulatory Elements

When developing adenoviral vectors for gene therapy, the viral mechanisms controlling transcription and translation are of particular importance, as they can enhance or reshape the expression of inserted transgenes. Incorporating these mechanisms into vector design can yield substantially higher expression levels than those achieved with isolated cellular promoters. The literature indicates that strong adenoviral promoters, such as the MLP and the E3 promoter, can efficiently initiate transgene transcription when the cassette is inserted in a parallel orientation relative to the direction of viral transcription. This makes it possible to harness late viral transcription programs to boost transgene expression. 

### 4.1. Major Late Promoter

The major late promoter plays a central role in adenoviral late transcription and is among the most powerful viral promoters known to function in mammalian cells [123,152]. Its activity is phase dependent: during the early stage of infection, transcription from the MLP is minimal, but after the onset of viral DNA replication, MLP-driven transcription increases markedly [153,154]. This increase depends, in part, on the early viral protein E1A, which functions as a transcriptional activator [155]. Transcription from the MLP produces a single long primary RNA, which is processed by alternative splicing to generate the late mRNA families L1–L5 encoding capsid structural proteins and other virion components [156].

A key feature of these transcripts is the tripartite leader, a three-exon 5′ untranslated sequence of approximately 200 nt present in all late adenoviral mRNAs [157]. TPL is generated by the splicing of three leader exons and plays a major role in post-transcriptional regulation: it promotes efficient export of mRNA from the nucleus to the cytoplasm and supports preferential translation of viral transcripts over cellular mRNAs [158]. This is mediated, in part, through interactions between TPL-containing mRNAs and early-phase adenoviral proteins from the E1 and E4 regions, E1B-55K and E4-orf6 [75,99]. These proteins form a specific nuclear complex: E1B-55K can associate with viral mRNA, while E4-orf6 facilitates export via signaling domains recognized by the nuclear export machinery [159]. In parallel, they inhibit export and translation of cellular mRNAs, including by reducing phosphorylation of the translation factor eIF2α [160]. Thus, adenovirus promotes expression of its own transcripts by redirecting host-cell resources toward viral protein synthesis. In E1-deleted vectors, however, some of these late-phase regulatory interactions may depend on the complementing cell line used for vector production and the availability of helper functions supplying E1 proteins in trans.

Studies of MLP and TPL indicate that transgene expression in adenoviral vectors is strongly influenced by these viral regulatory elements. Vectors carrying *GFP* in the forward orientation within the E3 locus under MLP control showed a stepwise increase in transcription as the number of TPL exons was increased, and the presence of E1 genes further enhanced expression—particularly in infected cells—consistent with activation of late transcription and synergy between viral factors [161]. Accordingly, transgenes inserted into the E3 locus under MLP control can be robustly expressed, especially when supported by adenoviral regulatory elements such as the TPL and E1 gene products. Other studies have also shown that the MLP contributes to balancing early and late gene expression: reduced MLP activity was associated with increased expression of early units (E1A, E1B, E2) and elevated expression of pIX, which lies outside the major late transcription unit (MLTU), underscoring the complex role of the MLP in controlling both viral and transgene expression [59].

### 4.2. The E3 Region Promoter

In addition to the MLP, the E3 region promoter represents a functional adenoviral regulatory element that can independently drive and enhance transgene expression [162]. Its activity is shaped by both viral factors—most notably E1A—and host transcriptional inputs. In mutant adenoviruses with impaired E3 promoter function, a short sequence element was identified that is critical for E1A trans-activation and contributes to both basal and inducible promoter activity [163]. Transfer of this motif into the E1A-insensitive herpes simplex virus tk promoter rendered it responsive to E1A, supporting the functional relevance of this regulatory module [164]. Notably, this element shows similarity to motifs found in promoters of other adenoviral early genes (including E2A and E4), suggesting partially shared mechanisms of transcriptional activation. Experimental models further indicate that the E3 promoter is highly responsive to cellular signaling. For example, T-lymphocyte activation cues (PMA plus ionomycin) increased E3 promoter-driven transcription by ~100-fold, exceeding the stimulation achieved by E1A expression alone (approximately 5-fold) [165]. This highlights the capacity of the E3 promoter to support strong yet signal-dependent expression.

The translational relevance of these properties has been demonstrated in oncolytic adenoviral platforms in which therapeutic genes were placed under E3 promoter control. High-level *GM-CSF* expression driven by the E3 promoter has been detected in vitro in tumor cells and in vivo in mouse serum and tumor tissues following vector administration [166]. Similarly, when *MCP-3* and *TNFα* were inserted into different E3 sites (6.7K/gp19K and ADP) and placed under E3 promoter control, transgene expression followed the characteristic temporal profile of E3 transcription, consistent with integration of the inserts into the native viral regulatory program. Importantly, endogenous E3 gene functions were preserved, and the virus retained replicative capacity and cytopathic activity [167]. Together, these findings illustrate that endogenous adenoviral promoters can be leveraged for therapeutic gene expression.

Overall, strong adenoviral promoters such as MLP and the E3 promoter can efficiently initiate transcription of inserted cassettes when the cassette orientation is aligned with the direction of viral transcription, particularly in E3-based configurations that also benefit from TPL- and E1-dependent regulatory inputs. This strategy can provide high transgene output by exploiting late-phase viral transcription and may be advantageous for applications requiring elevated protein production. Conversely, when tissue specificity or tight inducibility is required, cassette placement and orientation become critical design variables, because distinct genomic regions are embedded in different transcriptional programs and carry their own regulatory constraints. Therefore, insertion site selection and cassette direction should be guided by anticipated interactions with endogenous promoter activity and the architecture of adenoviral transcription units.

## 5. Conclusions

Transgene expression in adenoviral vectors is shaped not only by promoter choice but also by the transcriptional architecture of the viral genome, the insertion site and orientation, and interactions with endogenous viral regulatory elements. Inserted cassettes may become embedded within viral transcriptional programs, undergo alternative splicing, and be influenced by enhancer activity (including E1A) and RNA processing signals. Expression profiles depend on vector format: in single-cassette designs, the E1 region often supports robust expression, whereas in the E3 region, the orientation of insertion more strongly determines the extent to which the transgene is recruited into viral transcription. In dual-cassette vectors, additional risks arise from transcriptional interference and homologous recombination, necessitating careful selection of cassette orientations, spacing between expression units, and, where appropriate, the use of insulating elements. Endogenous adenoviral regulatory mechanisms, including the MLP and TPL, can be exploited to enhance expression via the late transcriptional program and preferential translation of TPL-containing mRNAs; however, this strategy inherently couples transgene activity to the infection phase. Therefore, rational adenoviral vector design should account for genomic context, insertion orientation, and the degree of cassette insulation, enabling the purposeful development of either high-output protein production systems or vectors with regulated or tissue-specific expression tailored to different gene therapy applications. Although many mechanistic insights summarized herein derive from studies using Ad5-based vectors, similar architectural principles are likely relevant to other adenoviral serotypes, although their transcriptional regulatory landscapes may differ. Nevertheless, these observations should be interpreted as general design guidelines rather than universal rules, as the magnitude and nature of these effects may vary depending on promoter choice, vector configuration, and cell type.

The authors of this review conclude that the literature synthesized herein supports a deeper understanding of how adenoviral transcriptional organization, insert orientation, and interactions with endogenous regulatory elements collectively determine the magnitude and pattern of transgene expression. Systematizing these findings provides a foundation for principles of rational adenoviral vector design—both for maximizing therapeutic protein output and for engineering precisely controlled or tissue-specific expression systems. Such an approach is essential for developing more effective and safer adenoviral platforms across a broad range of gene therapy applications.

## Figures and Tables

**Table 1:** Early Transcription Units of the Adenoviral Genome.

Early Transcription Units	Function	Reference
E1A	One of the earliest transcription units activated after the virus enters the nucleus. E1A proteins interact with cellular cell-cycle regulators (e.g., pRb) and initiate expression of other viral early genes. They drive the host cell into S phase, creating conditions for viral DNA replication.	[49,50,51,52,53]
E1B	Encodes the 19K and 55K proteins that prevent apoptosis induced either by the virus itself or by the host cell in response to E1A. In particular, the 55K protein interacts with p53 and blocks the apoptotic cascade.	[54,55,56,57,58]
E2A, E2B	Encode proteins directly involved in viral DNA replication: the DNA-binding protein (DBP), the viral DNA polymerase, and the preterminal protein (pTP), which participates in replication initiation at the 5′ end.	[59,60,61,62,63]
E3	A cluster of genes not required for replication but critical for immune evasion. E3 products reduce MHC-I expression on the cell surface, inhibit pro-inflammatory cytokines (e.g., TNFα), and suppress apoptosis triggered by extrinsic signals.	[64,65,66,67,68,69]
E4	Regulates late gene expression, transcription, mRNA transport, and the control of viral and cellular DNA damage responses. E4 proteins interact with cellular complexes such as E3 ubiquitin ligases and modulate DNA repair and gene expression.	[70,71,72,73,74,75]

## Data Availability

No new data were created or analyzed in this study. Data sharing is not applicable to this article.
